# Next Generation Sequencing (NGS) in chromosome translocation 46, XX, t (9; X) (q22; q28) - a case report

**DOI:** 10.5935/1518-0557.20180034

**Published:** 2018

**Authors:** Monise Santos, Ivan Henrique Yoshida, Caroline Zulim, Michelli Suemi Tanada, Emerson Barchi Cordts, Caio Parente Barbosa

**Affiliations:** 1 Instituto Ideia Fértil de Saúde Reprodutiva; 2 Faculdade de Medicina do ABC

**Keywords:** Next generation sequencing, chromosome translocation, assisted human reproduction, pre-implantation genetic diagnosis

## Abstract

This paper reports the case of a patient who sought assisted reproductive technology (ART) treatment and was referred to pre-implantation genetic diagnosis (PGD) on account of a chromosomal translocation presented with secondary infertility. The patient underwent a highly complex ART treatment and had 14 metaphase II oocytes collected on the day of follicular aspiration. The embryos were taken to extended culture and five were biopsied and vitrified. The embryo genetic report showed aneuploidy in four of the blastocysts, while the other resulted in 46, XX. In conclusion, chromosome translocations involving the X chromosome might result in the deregulation of gene expression and defective ovarian formation. Therefore, the genes present in the X chromosome are believed to be essential in normal ovarian function.

## INTRODUCTION

For decades embryo viability has been assessed based on embryo morphology (Ebner *et al.,* 2003), although many cycles do not result in gestation. Thus, it is believed that morphologically normal embryos might be aneuploid. In fact, some studies have shown that 50% of the formed blastocysts suffer from aneuploidy (Alfarawati *et al*., 2011; Goldman *et al*., 2016). However, the advancements in reproductive medicine have made it possible to evaluate embryo viability through chromosomal and gene mutation analysis.

In 1990, pre-implantation genetic diagnosis (PGD) was performed for the first time in a couple with genetic disease to rule out the possibility of transmitting the condition to their offspring (Harper, 2009). The technique is indicated to couples with known genetic or chromosomal alterations, with the purpose of preventing the transmission of the alterations to their offspring. Embryo biopsies may be performed in the zygote, cleavage or blastocyst stages. At present, most of the biopsies are performed in the blastocyst stage, since studies found that the rate of mosaicism in the cleavage stage is 50%, whereas in the blastocyst stage it is 3-5% (Munné *et al*., 1994; Brezina *et al*., 2013).

Chromosomal abnormalities include complete or partial deletions, the presence of an additional chromosome or translocations involving or not the sex chromosomes (Goswami & Conway, 2005). Chromosomal translocations occur because of a rearrangement between chromosomes and are 18% more likely to form euploid embryos. Cytogenetic abnormalities related to the X chromosome usually lead to early ovarian failure. A region of the long arm of the X chromosome known as "critical region Xq" - ranging from Xq13 to Xq28 - has been associated with the formation of the female gonad and ovarian function maintenance; therefore, the genes present in this chromosome are believed to be essential for normal ovarian function (Simpson & Rajkovic, 1999).

The most modern molecular technology used today is Next Generation Sequencing (NGS). The technique may be used to rule out chromosomal and genetic alterations, genetically analyze an embryo's twenty-four chromosomes with accuracy levels greater than 90%, identify losses and gains of genetic material, and examine aneuploidies of whole chromosomes and chromosomal trisomies. However, extremely small chromosomal changes, depending on the site of the chromosomal alteration, might not be detected by NGS.

## CASE REPORT

This is the case of a 29-year-old patient who had her menarche at the age of 12, with regular cycles lasting for four days, no history of gynecological surgery, and normal hormonal tests. Her partner was 28 years old and had normal hormonal tests and spermograms. The couple sought ART treatment at a fertility center (RHA) and was referred to PGD on account of chromosome translocation, 46, XX, t (9; X) (q22; q28).

Induction was performed with Pergoveris 150UI/75UI (rFSH/rLH), 0.25mg Cetrotide and Choriomon 5000IU. Fourteen metaphase II oocytes were harvested in follicular puncture and fertilized using the intracytoplasmic sperm injection technique (ICSI); semen analysis showed 3.9M, 96% progressive spermatozoa. The fertilization rate was 78.57% (11/14) and all embryos were taken to the blastocyst stage on extended culture.

Five blastocysts were biopsied and subsequently vitrified. The cells were removed from the trophectoderm, stored in appropriate solutions, and sent to the laboratory for embryo genetic analysis. NGS was used to analyze the 24 chromosomes.

The embryo genetic report revealed four aneuploid blastocysts (80%) and one euploid blastocyst (20%) (Table 1).

**Table 1:** Embryo genetic report results.

Nº Blastocyst	Embryo Genetic Analysis
1	47,XY,+9
2	45,XX,-13
3	45,XX,-2
4	45,XX,dup(2q),-9
5	**46,XX**

Endometrial preparation was performed with Primogyna 8mg/day and Utrogestan 600mg/day for later embryo transfer with a Sydney catheter. Ten days after embryo transfer, the BHCG test read 1,710 mIU/mL.

## DISCUSSION

Patients with known chromosome or gene anomalies and a track record of unsuccessful transfers undergoing ART treatment may resort to screening and genetic diagnostic tests to have embryos free of the assessed conditions transferred. In the present case report, the cycle of a patient with chromosomal translocation yielded a fertilization rate of 78.57% (11/14); embryo biopsies and molecular genetic tests with NGS were performed, leading to the transfer of a euploid embryo (20%).

Liss *et al*. (2015) studied 42 couples with reciprocal translocation and 35 with Robertsonian translocation through the FISH technique. The authors assessed fertilization rates, the number of biopsied embryos, and normal embryos. They observed that the group with reciprocal translocation had a fertilization rate of 70.8% versus 66.8% of the individuals with Robertsonian translocation. The overall fertilization rate was 68.9%. Five hundred and forty-eight embryos were biopsied and 101 euploid embryos were found, yielding a euploidy rate of 19.4%. A study carried out by our group examined nine patients with translocations identified with CGH-array and/or NGS tests and found fertilization and euploidy rates of 84% and 38%, respectively, similarly to Liss *et al*. (2015). Zhang *et al*. (2016) investigated 21 couples with history of translocation and miscarriages offered RHA cycles with PGD. Molecular genetic analysis was performed in 98 embryos, and a euploidy rate of 30.6% was found.

Chromosomal translocations alter the spatial organization of the "critical region Xq". PGD is of great importance in cases of genetic alterations to prevent the transmission of genetic disorders to the offspring. The age of the patient enabled the harvesting of a good number of oocytes, thus increasing the chance of finding a euploid blastocyst. In this cycle, in addition to having a healthy embryo transferred, the patient became pregnant.
